# Consideration of overadjustment bias in guidelines and tools for systematic reviews and meta-analyses of observational studies is long overdue

**DOI:** 10.1093/ije/dyad174

**Published:** 2023-12-21

**Authors:** Anita van Zwieten, Fiona M Blyth, Germaine Wong, Saman Khalatbari-Soltani

**Affiliations:** School of Public Health, Faculty of Medicine and Health, University of Sydney, Sydney, NSW, Australia; Centre for Kidney Research, Children’s Hospital at Westmead, Westmead, NSW, Australia; School of Public Health, Faculty of Medicine and Health, University of Sydney, Sydney, NSW, Australia; ARC Centre of Excellence in Population Ageing Research (CEPAR), University of Sydney, Sydney, NSW, Australia; School of Public Health, Faculty of Medicine and Health, University of Sydney, Sydney, NSW, Australia; Centre for Kidney Research, Children’s Hospital at Westmead, Westmead, NSW, Australia; Centre for Transplant and Renal Research, Westmead Hospital, Westmead, NSW, Australia; School of Public Health, Faculty of Medicine and Health, University of Sydney, Sydney, NSW, Australia; ARC Centre of Excellence in Population Ageing Research (CEPAR), University of Sydney, Sydney, NSW, Australia

**Keywords:** Systematic review, meta-analysis, overadjustment bias, confounding, guidelines, risk of bias, critical appraisal, observational studies, meta-research, bias

## Introduction

Systematic reviews and meta-analyses of observational studies are a common source of evidence used to inform clinical and health policy decisions. However, observational studies are inherently vulnerable to various biases, including selection, measurement and confounding biases. Therefore, it is critical to consider and properly address these sources of bias, to maximize the accuracy of the estimates that are obtained. When seeking to address bias, it is important to avoid introducing other types of bias.^1^ In particular, when seeking to address confounding bias in primary studies, it is common practice to mistakenly adjust for variables that lie on the causal pathway from the exposure to the outcome, which leads to overadjustment bias in estimation of total effects.^1^^,^^2^ Overadjustment is likely to result in bias towards the null for the total effect of the exposure on the outcome of interest, as it closes some causal paths and may also lead to collider stratification bias, which can result in bias in any direction (underestimation or overestimation of the effect of interest).^1^^,^^2^

There is an extensive body of guidelines and standards for conducting and reporting systematic reviews, alongside a large number of tools for evaluating the risk of bias (ROB) of included studies.^3^ Guidelines play an important role in researchers’ awareness of the importance of rigorous conduct and which items must be reported in systematic reviews, and ROB tools are used to assess, systematically report on and manage ROB for included studies (e.g. through sensitivity analyses, interpretation and discussion of results).^3^^,^^4^ However, the existence of guidelines and tools is not enough, given that the uptake of and adherence to guidelines and use of ROB tools are known to be suboptimal. For instance, a meta-research study on 300 systematic reviews of biomedical research^5^ found that, despite an overall improvement in reporting for 2014 reviews compared with those published in 2004, the reporting of many included reviews was still poor and one-third of them did not report using ROB tools. Similarly, a review of meta-research studies on adherence to the Preferred Reporting Items for Systematic Reviews and Meta-Analyses (PRISMA) statement and its extensions^6^ found suboptimal adherence overall. There is some evidence that guidelines improve reporting, with self-reported use of the PRISMA Statement or Cochrane methodology in reviews associated with greater reporting completeness.^5^

Systematic review guidelines and tools commonly include an emphasis on key domains of confounding, selection and measurement biases.^4^^,^^7^ Given their potential positive influence on conduct and reporting, it is important that systematic review guidelines, standards and ROB tools address all relevant sources of bias to guide reviewers, including overadjustment. To our knowledge, there has not been an evaluation of whether and to what extent overadjustment bias is considered in commonly used guidelines, standards and ROB tools for systematic reviews and meta-analyses of observational studies. We therefore aimed to provide an overview of the extent to which overadjustment bias has been considered in guidelines, standards and ROB tools in these settings. As a secondary aim, we compared the consideration of overadjustment bias with consideration of confounding bias, given that two types of bias are intrinsically linked and need to be considered together when deciding what to adjust for in study design and analysis. We hypothesized that overadjustment would rarely be explicitly considered in existing tools and guidelines, and that consideration of confounding would be greater than that of overadjustment bias.

## Methods

We examined key guidelines and standards for conducting and reporting systematic reviews and meta-analyses (e.g. handbooks, guides, reporting guidelines, conduct guidelines) and ROB/critical appraisal/quality assessment tools for individual studies in systematic reviews and meta-analyses. The focus was on general guidelines and tools relevant to observational studies of aetiology, prognosis and interventions, not on randomized controlled trials (RCTs), for which overadjustment bias is less relevant. We focused on common observational designs including cohort, cross-sectional and case-control designs and excluded those focused on other types of studies (e.g. diagnostic test accuracy studies, case studies, case series and studies of measurement properties). Inclusion was limited to generic guidelines and tools, with those that are specific to particular fields or population groups (e.g. health equity, psychology, dietetics, environmental research) being excluded. We examined those published from 2005 onwards, as key papers on overadjustment bias were published around this time,^2^^,^^8^ and focused on tools and guidelines developed for use across reviews (rather than those developed by authors for use in their own specific review). Frameworks for grading levels of evidence were excluded, as this was beyond our scope.

A multifaceted approach was used to identify relevant tools and guidelines, including those identified in previous reviews and meta-research studies on ROB tools^5^^,^^7^^,^^9–13^ and guidelines^3^; the PRISMA, EQUATOR network, Joanna Briggs Institute and Cochrane websites for guidelines; and expert knowledge. For included guidelines and tools, we extracted information on the scope, any consideration of overadjustment bias and any consideration of confounding bias, including relevant excerpts from the text or checklists. We then classified consideration of overadjustment and confounding (separately) as Yes/No/Some relevant guidance. In order to be classified as ‘Yes’, the tool/guideline needed to explicitly consider confounding/overadjustment, whereas ‘Some relevant guidance’ was used where the tool/guideline did not discuss the bias in detail but provided some guidance that would be relevant to addressing this type of bias. For instance, if the tool/guideline did not explicitly mention overadjustment bias but provided guidance on what constitutes a confounder and highlighted explicitly that adjusting for variables that lie on the causal pathway or post-intervention variables is not appropriate, we used the ‘Some relevant guidance’ classification for consideration of overadjustment bias, as they implicitly provided the necessary information to avoid overadjustment.

## Results

**Figure 1.**
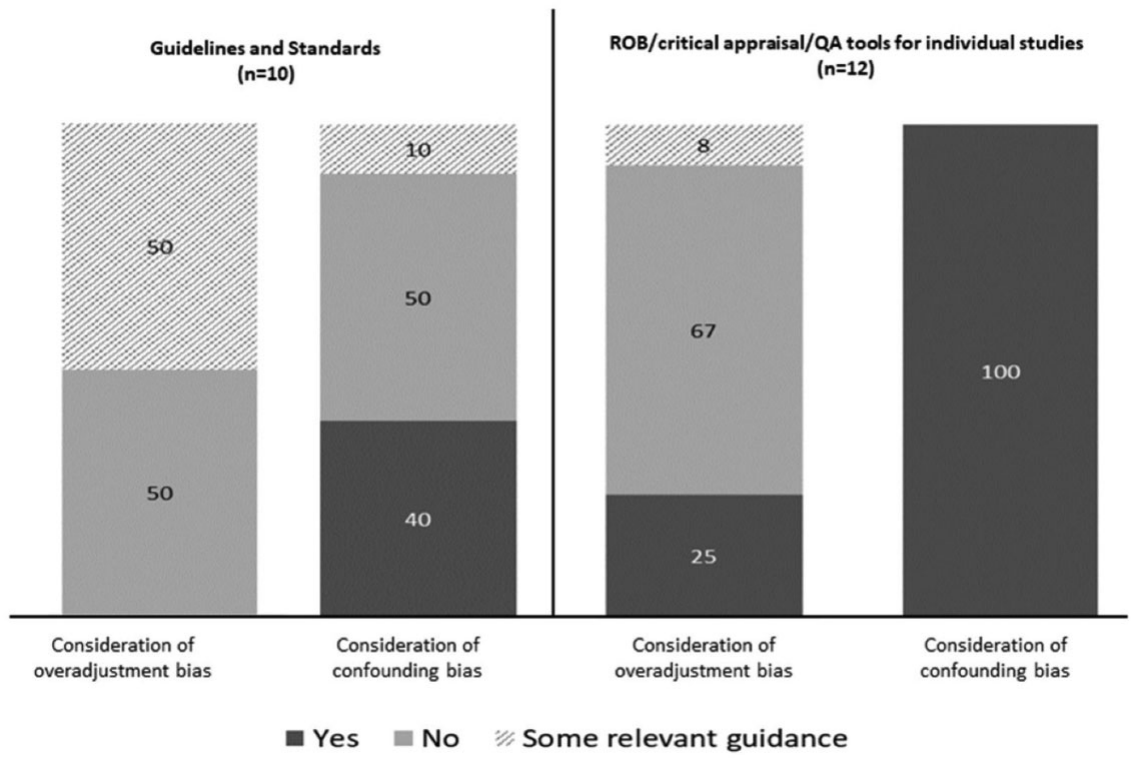
Consideration of overadjustment bias and confounding bias in included guidelines and standards for systematic reviews and meta-analyses (*n *= 10) and ROB/critical appraisal/QA tools for individual studies in systematic reviews and meta-analyses (*n* = 12). ROB, risk of bias; QA, quality assessment. Results are expressed as percentages

We identified 10 guidelines and 12 ROB tools (or suites of tools) for individual studies. Detailed information on the scope, consideration of overadjustment and consideration of confounding for each guideline and tool is provided in Supplementary Table S1 (available as Supplementary data at *IJE* online) and results for consideration of overadjustment and confounding biases are illustrated in Figure 1. For the guidelines, 4/10 received a Yes classification for explicitly discussing confounding, and none (0/10) explicitly considered overadjustment. Of the guidelines, 5/10 provided some relevant guidance on overadjustment bias (e.g. by encouraging authors to explicitly consider causal pathways between interventions/exposures and outcomes, stating that confounders should not be on the causal pathway from the exposure to the outcome, or by recommending the use of ROB assessment tools that consider overadjustment bias), and 1/10 provided some relevant guidance on confounding. For the ROB/critical appraisal/quality assessment tools, 12/12 received a Yes classification for explicitly considering confounding bias, and only 3/12 explicitly considered overadjustment bias. One tool (1/12) provided some relevant guidance on overadjustment bias, by encouraging reviewers to tailor their confounding bias assessment based on conceptual models and to consider whether the statistical model building was based on a conceptual framework.^14^

## Discussion

Overall, overadjustment bias has rarely been explicitly considered in existing guidelines for systematic reviews and meta-analyses of observational studies (0 out of 10; 0%) and tools for appraising ROB of individual studies in reviews (3 out of 12; 25%). In contrast, confounding was much more frequently and appropriately addressed, being explicitly considered in 40% (4 out of 10) of guidelines and 100% (12 out of 12) of ROB tools. ROB tools that explicitly addressed overadjustment bias (ROBINS-I,^15^ ROBINS-E^16^ and the confounder matrix^17^) were published recently, from 2016 to 2022, suggesting there has been an increase in awareness of this topic over time. We found that 50% (5 out of 10) of guidelines and 8% (1 out of 12) of ROB tools provided some relevant guidance for overadjustment, notably by discussing the importance of using logic models and conceptual frameworks or considering causal pathways when defining confounders.

This is the first attempt to understand the extent to which guidelines, standards and ROB tools for systematic reviews and meta-analyses of observational studies considered overadjustment bias in comparison with confounding bias considerations. Our findings align with problems that we have identified in the literature through our previous meta-research scoping review on approaches to overadjustment bias in systematic reviews and meta-analyses in the area of health inequities. Across 84 reviews, we found that approaches to managing overadjustment were not regularly applied. For example, <5% of reviews constructed causal diagrams, only 5% clearly defined both confounders and mediators, and whereas 54% of reviews considered confounding bias in their ROB assessment, only 2% had considered overadjustment in their ROB assessment.^18^ Although our scoping review focused on the example of health inequities studies, our findings are also relevant to reviews across other areas of epidemiology.

This is a novel topic with important implications for improving the conduct and reporting of systematic reviews and meta-analyses of observational studies, but there are some potential limitations and challenges. Categorizing the level of consideration of overadjustment bias was challenging in some cases because guidelines, standards and tools provided variable levels and types of guidance. For example, some discussed related issues without explicitly mentioning overadjustment (e.g. considering causal pathways) and these were labelled as ‘some relevant guidance’. We acknowledge that a number of the guidelines and tools was developed with a focus on interventions, particularly in the context of RCTs (e.g. Cochrane guidelines), and this may explain why overadjustment and/or confounding bias were not explicitly discussed. Guidelines, standards and ROB tools for RCTs may consider confounding through recommendations on randomization methods and allocation concealment, without explicitly mentioning confounding, which we did not capture. Because we limited our scope to guidelines and tools published after 2005 to capture the period after publication of key overadjustment papers, we did not include some key guidelines (e.g. Meta-Analysis of Observational Studies in Epidemiology: MOOSE^19^) and tools that have been very commonly used in the literature^7^^,^^9^^,^^10^ (e.g. the Newcastle Ottawa Scales^20^ and Downs and Black tool^21^)—regardless, none of these considered overadjustment bias. Similarly, we excluded discipline-specific tools, and some of these do address overadjustment. The PRISMA-Equity extension^22^^,^^23^ provides some relevant guidance by recommending the use of logic models or analytical frameworks and encouraging consideration of causal pathways and mechanisms of intervention impacts. The American Psychological Association reporting guidelines for meta-analysis (MARS)^24^ recommend discussing the rationale for the selection and coding of mediators, defining the coding categories for mediators, reporting results of mediator analyses and reporting interrelations among variables used for moderator and mediator analyses.

Systematic reviews and meta-analyses are key sources of evidence informing clinical and policy decisions, and therefore it is critical that they are conducted and reported using robust methods to provide accurate estimates with minimal bias. Thus, using review guidelines and ROB tools, review authors should seek to minimize bias. The harmful effect of overadjustment bias on precision and its relevance to all epidemiological research^2^^,^^25–29^ highlights the need for prioritization and explicit consideration of overadjustment bias in review guidelines and tools—despite the generally smaller magnitude of the impact from this bias compared with confounding bias.^30^ In addition, in light of our findings that overadjustment has not been explicitly considered in guidelines and standards, and has only been explicitly considered in more recent ROB tools, we believe that this issue should be given explicit consideration in new revisions of guidelines for systematic reviews and meta-analyses moving forward. Further, review authors should be encouraged to adopt the more recent ROB tools for primary studies which do explicitly consider overadjustment bias (e.g. ROBINS-I^15^, ROBINS-E^16^). More broadly, as identified elsewhere,^10^ there is a need to ensure that guidelines are tailored to reviews that include observational studies, including addressing the types of bias (notably overadjustment bias and confounding bias) that are particularly relevant to observational studies compared with RCTs. Given that uptake and adherence to guidelines and tools are suboptimal,^5^^,^^6^ multifaceted actions are needed to address this across all stages of research and from all relevant stakeholders (including researchers, editors, peer reviewers, publishers, ethics committees, universities and funders).^31^ As a first step, journals could require the use of guidelines and tools that address relevant types of bias for submissions, with appropriate flexibility to accommodate the complexity of epidemiological study design and variation in the relevance of individual criteria. Critically, rather than being overly rigid and prescriptive, the emphasis should be on promoting guidelines and tools as devices to enhance the quality of thinking that informs research design and interpretation, promote transparency of assumptions and facilitate robust discussion in the scientific community. As an example of the complexity of these issues, it can at times be difficult to determine whether a variable is best identified as a confounder or a mediator. For example, a variable may act as a confounder if it occurs before the exposure, or a mediator if it occurs after the exposure, but measurements may not be available at both time points in the dataset (or we may only have a measure that is concurrent with the exposure, which adds more ambiguity). It may also be challenging to determine whether a variable is best considered a mediator, due to lack of knowledge on the causal pathways underlying the effect of the exposure. In these situations, several strategies can be employed, including drawing a causal diagram depicting each variable at all relevant time points (e.g. physical activity at Time 1, Time 2, Time 3), carefully considering: timing of measurement and occurrence of all variables; reflecting existing knowledge on mechanisms and causal pathways; and conducting and presenting sensitivity analyses that treat a variable as a confounder and then as a mediator, with assumptions of each analysis clearly described.^1^^,^^32^

These recommendations are relevant for both individual studies and systematic reviews and meta-analyses. In addition, it is critical that overadjustment bias is embedded into research training and teaching curricula in epidemiology, health and medicine alongside confounding bias, in order to ensure that researchers and those who are implementing evidence in clinical practice have a sound understanding of both of these issues alongside other types of bias. It may be helpful to introduce the concept of Table 2 fallacy, which occurs when researchers present effect estimates for secondary variables (e.g. confounders and effect modifiers) in an epidemiological model (commonly in a single Table 2), which encourages readers to interpret all estimates as total-effect estimates, when in reality the model was built only to estimate the total effect of the main exposure.^33^ Estimates for secondary variables in the model in these circumstances may be subject to residual confounding, overadjustment and/or collider bias.

## Conclusion

We therefore recommend a multifaceted approach to overadjustment bias, including: explicit inclusion in guidelines and standards; encouraging uptake of recent tools that address overadjustment and confounding; and implementing strategies to improve uptake and adherence to guidelines, from educating students and researchers through to journal peer review and publication processes.

## Ethics approval

Ethics approval was not required for this project as it does not involve primary data.

## Supplementary Material

dyad174_Supplementary_Data

## Data Availability

All data are available in the Supplementary File (available as Supplementary data at *IJE* online).
