# Supplementary material for: Consideration of overadjustment bias in guidelines and tools for systematic reviews and meta-analyses of observational studies is long overdue
Source: Int J Epidemiol. 2023 Dec 21;53(1):dyad174. doi: 10.1093/ije/dyad174 (PMC10859154; doi:10.1093/ije/dyad174)
Supplement: dyad174_Supplementary_Data [file dyad174_supplementary_data.docx]

# SUPPLEMENTARY MATERIAL

## Table S1. Consideration of confounding and overadjustment bias in commonly used guidelines, standards, and risk of bias/critical appraisal/quality assessment tools for systematic reviews and meta-analyses of observational studies of aetiology, prognosis, and interventions

|  | **Consideration of overadjustment bias** | |  | **Consideration of confounding bias** | | **Notes on scope of guideline/**  **standard/**  **tool** |
| --- | --- | --- | --- | --- | --- | --- |
| **Guides and Standards** | | | | | | |
| Centre for Reviews and Dissemination (CRD) Guidance for Undertaking Reviews in Health Care^1^ (2009) | Some relevant guidance | Recommendation to establishing a theoretical basis (mechanism of action – causal chain) or framework for the intervention (chapters 1, 3, 4, and 6) as the first step of the review. Overadjustment not explicitly mentioned in any of the chapters. |  | Yes | Chapter 1 (general recommendations for systematic reviews) recommends consideration of confounding in risk of bias assessment of RCTs and observational studies.  Chapter 2 (reviews of clinical tests) – specific recommendations for consideration of confounding bias in quality assessment and data synthesis in prognostic systematic reviews.  Chapter 3 (reviews of public health interventions) – recommendation on establishing a theoretical basis for the intervention and consideration of the context. Confounding not explicitly mentioned.  Chapter 4 (reviews of adverse effects)– specific recommendations for consideration of confounding bias in assessing the generalisability of studies in systematic reviews of adverse effects.  Chapter 5 and 6: Confounding not explicitly mentioned. | Conduct guideline. Focused on interventions. |
| Cochrane Handbook for Systematic Reviews of Interventions (2022)^2^ | Some relevant guidance | **Part 1:** Chapter 3 has general recommendations relating to ROB including assessment, reporting, and considerations in synthesis and discussion. No explicit mention of overadjustment bias, though recommends use of ROBINS-I for NRSI which includes overadjustment bias. In the guidance for introductions, there is explicit consideration of causal pathways through which interventions may impact the outcome and suggestion of use of logic models/conceptual frameworks.  Other chapters of Part 1 do not explicitly mention overadjustment bias.  **Part 2**: Not considered for this table as the focus is on RCTs.  **Part 3**: No explicit mention of overadjustment bias. Similar to Chapter 3, in Chapters 16 and 17, there is explicit consideration of causal pathways through which interventions may impact the outcome and suggestion of use of logic models/conceptual frameworks.  **Part 4**: Not considered explicitly in guidance, but in Chapter 25 on assessing ROB in NRSI, use of ROBINS-I^3^ is recommended, and ROBINS-I considers overadjustment bias. |  | Yes | **Part 1:** Chapter 3 has general recommendations relating to ROB including assessment, reporting, and considerations in synthesis and discussion. No explicit mention of confounding, though recommends use of ROBINS-I for NRSI which includes confounding.  Other chapters of Part 1 do not explicitly mention confounding bias.  **Part 2**: Not considered for this table as the focus is on RCTs.  **Part 3**: Some brief mention of confounding.  **Part 4:** Chapters 24-25 on NRSI have a strong focus on addressing confounding throughout review stages e.g., review authors should attempt to pre-specify important confounding domains in their protocol if NRSI are being included, prioritise extraction of adjusted estimates for NRSI to minimise confounding. | Reporting and conduct guideline. Focused on interventions, mainly on randomised studies (but Chapters 24-25 focus on NRSI). |
| COSMOS-E: Guidance on conducting systematic reviews and meta-analyses of observational studies of etiology^4^ (2019) | Some relevant guidance | No explicit mention of considering overadjustment bias. However, when considering matching as one of the approaches to deal with confounding in observational studies, the authors mention: “The choice of the variables, exact approach to matching, and the statistical analysis are complex and need careful consideration. Bias may be introduced, for example, when matching for variables that are on the causal pathway from exposure to disease.” |  | Yes | Recommends consideration of confounding in review planning, writing protocol, data extraction, ROB assessment, result synthesis, meta-analysis, and interpretation and discussion of results.  Signalling questions for ROB include:   - “What are the important variables that might confound the effect of the exposure?” - “Were these variables measured with precision and at appropriate points in time?” - “Did the authors use an appropriate analysis method or design that adjusted for all of the important confounding variables?” | Conduct guideline. Focused on observational studies of etiology. |
| Joanna Briggs Institute (JBI) Manual for Evidence Synthesis^5^ – specifically Chapter 7 on Systematic Reviews of Etiology and Risk (2020) | Some relevant guidance | When defining confounding, manual states: “Confounding occurs when another factor other than primary factor of interest or being investigated, can directly influence the outcome being measured. To be classed as a confounding factor, it should not be a factor that appears in the casual pathway between and exposure and the outcome.” Overadjustment is not included in the JBI critical appraisal checklists as outlined below. |  | Yes | Specific guidance on defining confounding, and considering confounding in data extraction, result synthesis, and meta-analysis. Confounding is included in the JBI critical appraisal checklists as outlined below. | Conduct and reporting guideline. Chapter 7 is focused on reviews of etiology and risk. |
| Methodological Expectations of Cochrane Intervention Reviews (MECIR) (2022)^6^ | No | General recommendations relating to ROB including assessment, reporting, and considerations in synthesis and discussion. No explicit mention of overadjustment. Refers readers to extensive guidance in Cochrane Handbook and online training resources which may provide further relevant guidance. |  | No | General recommendations relating to ROB including assessment, reporting, and considerations in synthesis and discussion. No explicit mention of confounding. Refers readers to extensive guidance in Cochrane Handbook and online training resources which may provide further relevant guidance. | Reporting and conduct standards for Cochrane reviews. Focused on interventions, mainly on randomised studies. |
| PRISMA 2020 Statement: an updated guideline for reporting systematic reviews^7, 8^ (2021) | No | General recommendations relating to ROB including assessment, reporting, and consideration in synthesis. |  | No | General recommendations relating to ROB including assessment, reporting, and consideration in synthesis. Some brief mention of confounding in explanation and elaboration document^8^. | Reporting guideline. Focused on interventions; applicable to other contexts. |
| PRISMA-IPD 2015: Preferred Reporting Items for a Systematic Review and meta-analysis of Individual Participant Data^9^ (2015) | No | General recommendations relating to ROB including assessment, reporting, and synthesis. Overadjustment not explicitly mentioned. |  | No | General recommendations relating to ROB including assessment, reporting and synthesis. Confounding not explicitly mentioned. | Reporting guideline. Focused on randomized trials; applicable to other contexts. |
| PRISMA-P Statement 2015: Preferred Reporting Items for Systematic Review and Meta-Analysis Protocols^10, 11^ (2015) | No | General recommendations relating to ROB including assessment, reporting, and synthesis. Overadjustment not explicitly mentioned. |  | No | General recommendations relating to ROB including assessment, reporting and synthesis. Confounding mentioned in relation to GRADE evidence certainty. | Reporting guideline. Focused on interventions. |
| RAMESES publication standards: realist syntheses^12^ (2013) | Some relevant guidance | Recommendation to consider interaction between context, mechanism, and outcome in explaining the focus of review, methods (e.g., data extraction, analysis, and data synthesis), and main findings. Overadjustment not explicitly mentioned. |  | Some relevant guidance | Recommendation to consider interaction between context, mechanism, and outcome in explaining the focus of review, methods (e.g., data extraction, analysis, and data synthesis), and main findings. Confounding not explicitly mentioned. | Reporting guideline. Focused on realist synthesis. |
| Synthesis without meta-analysis (SWiM) in systematic reviews: reporting guideline^13^ (2020) | No | General recommendations relating to ROB including assessment, reporting, and synthesis. Overadjustment not explicitly mentioned. |  | No | General recommendations relating to ROB including assessment, reporting and synthesis. Confounding not explicitly mentioned. | Reporting guideline. Focused on interventions. |
| **Risk of bias/critical appraisal/quality assessment tools for individual studies included in systematic reviews and meta-analyses** | | | | | | |
| Critical Appraisal Skills Programme (CASP) Checklists for Cohort Studies and Case-Control Studies^14^ (2018) | No | No explicit mention of overadjustment. |  | Yes | Several checklist items relate to confounding:   - Case-control checklist: “Aside from the experimental intervention, were the groups treated equally?” Cohort checklist: “Have the authors identified all important confounding factors?” - Both checklists: “Have the authors taken account of the potential confounding factors in the design and/or analysis?” - Case-control checklist: “How large was the treatment effect? Consider: what are the bottom line results, is the analysis appropriate to the design, how strong is the association between exposure and outcome (look at the odds ratio), are the results adjusted for confounding and might confounding still explain the association, has adjustment made a big difference to the OR.” - Both checklists: “Do you believe the results? Consider: big effect is hard to ignore, can it be due to chance, bias, or confounding, are the design and methods of this study sufficiently flawed to make the results unreliable, Bradford Hills criteria.” | Focused on cohort and case-control studies. Designed as educational pedagogical tools. |
| Effective Public Health Practice Project (EPHPP) Quality Assessment Tool for Quantitative Studies^15-17^ (2010) | No | Dictionary includes a definition of confounders that does not exclude mediating variables: “By definition, a confounder is a variable that is associated with the intervention or exposure and causally related to the outcome of interest. Even in a robust study design, groups may not be balanced with respect to important variables prior to the intervention. The authors should indicate if confounders were controlled in the design (by stratification or matching) or in the analysis. If the allocation to intervention and control groups is randomized, the authors must report that the groups were balanced at baseline with respect to confounders (either in the text or a table).” |  | Yes | One domain of the tool relates to confounding:   - “Were there important differences between groups prior to the intervention? The following are examples of confounders: 1. Race, 2. Sex, 3. Marital status/family, 4. Age, 5. SES (income or class), 6. Education, 7. Health status, 8. Pre-intervention score on outcome measure.” - “If yes, indicate the percentage of relevant confounders that were controlled (either in the design (e.g. stratification, matching) or analysis).” | Focused on interventions. |
| Joanna Briggs Institute (JBI) critical appraisal checklists for cohort studies, case-control studies and analytical cross-sectional studies^5^ (2020) | No | While the JBI manual excludes variables on the causal pathway from the definition of confounders, the checklist guidance does not: “Confounding has occurred where the estimated intervention exposure effect is biased by the presence of some difference between the comparison groups (apart from the exposure investigated/of interest). Typical confounders include baseline characteristics, prognostic factors, or concomitant exposures (e.g. smoking). A confounder is a difference between the comparison groups and it influences the direction of the study results…” |  | Yes | All checklists include the following considerations for confounding:   - “Were confounding factors identified?” - “Were strategies to deal with confounding factors stated?” - “Was appropriate statistical analysis used?” (which includes considerations related to confounding per the guidance document). | Focused on systematic reviews of aetiology and risk (from JBI manual Chapter 7). |
| Mixed Methods Appraisal Tool (MMAT) version 2018^18^ (2018) | No | Explanatory notes include a definition of confounding that does not explicitly exclude mediating variables: “Confounders are factors that predict both the outcome of interest and the intervention received/exposure at baseline.” |  | Yes | Appraisal question on confounding for quantitative non-randomized studies:   - “Are the confounders accounted for in the design and analysis?” | Focused on systematic mixed studies reviews (reviews that include qualitative, quantitative and mixed methods studies). Designed to appraise the methodological quality of qualitative research, RCTs, non-randomized studies, quantitative descriptive studies, and mixed methods studies. |
| National Heart Lung and Blood Institute (NHLBI) Quality Assessment Tool for Observational Cohort and Cross-Sectional Studies and Tool for Case Control Studies^19^ (2013) | No | Guidance includes definitions of confounding that do not exclude mediating variables:   - “This is a key issue in case-controlled studies; statistical analyses need to control for potential confounders, in contrast to RCTs in which the randomization process controls for potential confounders. In the analysis, investigators need to control for all key factors that may be associated with both the exposure of interest and the outcome and are not of interest to the research question. A study of the relationship between smoking and CVD events illustrates this point. Such a study needs to control for age, gender, and body weight; all are associated with smoking and CVD events. Well-done case-control studies control for multiple potential confounders.” - “This is a key issue in cohort studies, because statistical analyses need to control for potential confounders, in contrast to an RCT, where the randomization process controls for potential confounders. All key factors that may be associated both with the exposure of interest and the outcome–that are not of interest to the research question–should be controlled for in the analyses. For example, in a study of the relationship between cardiorespiratory fitness and CVD events (heart attacks and strokes), the study should control for age, BP, blood cholesterol, and body weight, because all of these factors are associated both with low fitness and with CVD events. Well-done cohort studies control for multiple potential confounders.” - “Critical appraisal involves considering the risk of potential for selection bias, information bias, measurement bias, or confounding (the mixture of exposures that one cannot tease out from each other). Examples of confounding include co-interventions, differences at baseline in patient characteristics, and other issues throughout the questions above.” |  | Yes | One checklist item on confounding in each tool:   - Cohort/Cross-Sectional Tool: “Were key potential confounding variables measured and adjusted statistically for their impact on the relationship between exposure(s) and outcome(s)?” - Case-Control Tool: “Were key potential confounding variables measured and adjusted statistically in the analyses? If matching was used, did the investigators account for matching during study analysis?”   Confounding is also highlighted in the guidance on overall quality ratings. | Focused on observational cohort and cross-sectional studies, and case-control studies, respectively. |
| National Institute for Health and Care Excellence (NICE) quality appraisal checklist for quantitative studies reporting correlations and associations and quality appraisal checklist for quantitative intervention studies^20^ (2012) | No | No explicit mention of overadjustment bias. |  | Yes | Several items in the checklists relate to confounding.  In the checklist for studies reporting correlations and associations:  Method of selection of exposure group:   - “How well were likely confounding factors identified and controlled?   - Were there likely to be other confounding factors not considered or appropriately adjusted for?   - Was this sufficient to cause important bias?”   Analyses:   - “Were the analytical methods appropriate?   - Were important differences in follow-up time and likely confounders adjusted for?”   Summary:   - “Are the study results internally valid (i.e. unbiased)?   - How well did the study minimise sources of bias (i.e. adjusting for potential confounders)?   - Were there significant flaws in the study design?”   In the checklist for quantitative intervention studies:  Method of allocation to intervention (or comparison):   - “Allocation to intervention (or comparison). How was selection bias minimised?   - Was allocation to exposure and comparison randomised? Was it truly random ++ or pseudo-randomised + (e.g. consecutive admissions)?   - If not randomised, was significant confounding likely (−) or not (+)?”   Analyses:   - “Were exposure and comparison groups similar at baseline? If not, were these adjusted?   - Were there any differences between groups in important confounders at baseline?   - If so, were these adjusted for in the analyses (e.g. multivariate analyses or stratification).   - Were there likely to be any residual differences of relevance?” - “Were the analytical methods appropriate?   - Were important differences in follow-up time and likely confounders adjusted for?”   Summary:   - “Are the study results internally valid (i.e. unbiased)?   - How well did the study minimise sources of bias (i.e. adjusting for potential confounders)?   - Were there significant flaws in the study design?” | Focused on public health. Checklist for quantitative intervention studies is designed to be used for RCTs, case-control studies, cohort studies, controlled before-and-after studies and interrupted time series. |
| Quality in Prognosis Studies Tool (QUIPS)^21^ (2013) | Some relevant guidance | No explicit mention of overadjustment. However, authors are encouraged to tailor the confounding assessment to their research question including a suggestion to identify key variables “in a conceptual model”. Further, the statistical analysis and reporting domain includes as a consideration: “Strategy for model building is appropriate and is based on a conceptual framework or model”. |  | Yes | One domain focused on confounding with multiple factors to consider in evaluating the risk of bias due to confounding as high, moderate or low:  “a. All important confounders are measured  b. Clear definitions of the  important confounders measured are provided  c. Measurement of all important confounders is adequately valid and reliable  d. The method and setting of confounding measurement are the same for all study participants  e. Appropriate methods are used if imputation is used for missing confounder data  f. Important potential confounders are accounted for in the study design  g. Important potential confounders are accounted for in the analysis” | Focused on studies of prognostic factors. |
| Risk of Bias Assessment Tool for Non-randomized Studies (RoBANS)^22^ (2013) | No | No mention of overadjustment. |  | Yes | One domain focused on confounding, including the following for low risk of bias judgements:   - “The major confounding variables were adequately confirmed and considered during the design phase (e.g., through matching, participation restriction, or other methods). - The major confounding variables were adequately confirmed and adjusted for during the analysis phase (e.g., through stratification, propensity score approaches, statistical adjustments, or other methods).” | Focused on non-randomised studies of interventions. |
| ROBINS-I: a tool for assessing Risk of Bias in Non-Randomised Studies of Interventions^3^ (2016) | Yes | Confounding criteria include explicit consideration of overadjustment:  “Did the authors control for any post-intervention variables that could have been affected by the intervention? Controlling for post-intervention variables that are affected by intervention is not appropriate. Controlling for mediating variables estimates the direct effect of intervention and may introduce bias. Controlling for common effects of intervention and outcome introduces bias.” |  | Yes | Confounding incorporated from the protocol stage through identification of relevant confounding domains. When assessing ROB, ROBINS-I includes:   - Preliminary consideration of relevant confounders including confounding domain, measured variables, whether controlling for excluded variables was unnecessary, whether the domain was measured validly and reliably by the variables, expected direction of bias. - One domain of bias assessment dedicated to confounding, with multiple signalling questions to address baseline confounding and time-varying confounding.   Signalling questions include:   - “Is there potential for confounding of the effect of intervention in this study?” - “Did the authors use an appropriate analysis method that controlled for all the important confounding domains and for time-varying confounding?” - “Were confounding domains that were controlled for measured validly and reliably by the variables available in this study?” - “What is the predicted direction of bias due to confounding?” | Focused on non-randomised studies of interventions. |
| Risk Of Bias In Nonrandomized Studies - of Exposure (ROBINS-E) for follow-up (cohort) studies - Launch Version^23^ (2022) | Yes | Confounding criteria include explicit consideration of overadjustment:   - Baseline confounding: “Did the authors control for any variables after the start of the exposure period being studied that could have been affected by the exposure? Controlling for post-exposure variables that are affected by exposure is not appropriate (this is sometimes called ‘over-adjustment’). Controlling for mediating variables estimates the direct effect of exposure and may introduce bias. Controlling for common effects of exposure and outcome (sometimes referred to as ‘colliders’) introduces selection bias.” - Baseline and time-varying confounding: “Did the authors control for time-varying factors or other variables measured after the start of the exposure window being studied? This question is asked if an inappropriate analysis method has been used to control for time-varying confounding factors. In such a situation, controlling for (conditioning on) factors measured after the start of the exposure window is likely to lead to bias because these are also on the causal pathway from the exposure to the outcome.” |  | Yes | Confounding incorporated from the protocol stage through identification of relevant confounding domains. When assessing ROB, ROBINS-E includes:   - Consideration of whether association is at such high ROB due to confounding that further assessment is unnecessary. - Preliminary consideration of relevant confounders including confounding domain, measured, and adjusted variables, whether controlling for excluded variables was unnecessary, whether the domain was measured validly and reliably by the variables, expected direction of bias. - One domain of bias assessment dedicated to confounding, with signalling questions to address baseline and time-varying confounding.   Signalling questions include:   - “Did the authors use an analysis method that was appropriate to control for time varying as well as baseline confounding?” - “Did the authors control for all the important baseline and time-varying confounding factors for which this was necessary?” - “Were confounding factors that were controlled for (and for which control was necessary) measured validly and reliably by the variables available in this study?” - “Did the use of negative controls, or other considerations, suggest uncontrolled confounding?” - “Risk of bias (due to confounding) in the estimated effect of exposure on the outcome” - “What is the predicted direction of bias due to confounding?” - “Is the risk of bias (due to confounding) sufficiently high, in the context of its likely direction and the magnitude of the estimated exposure effect, to threaten conclusions about whether the exposure has an important effect on the outcome?” | Focused on cohort studies examining effects of exposure on outcome. |
| Scottish Intercollegiate Guidelines Network (SIGN) checklist for cohort studies v3 and checklist for case-control studies v2^24^ (2012) | No | Guidance includes a definition of confounding that does not exclude mediating variables: “Confounding is the distortion of a link between exposure and outcome by another factor that is associated with both exposure and outcome…Clinical judgement should be applied to consider whether all likely confounders have been considered.” |  | Yes | The Methodology checklists for cohort and case-control studies include the following considerations for confounding:   - “The main potential confounders are identified and taken into account in the design and analysis.” - “How well was the study done to minimise the risk of bias or confounding?” | Focused on cohort and case-control studies. Used for systematic reviews that inform guideline development. |
| The Confounder Matrix^25^ (2022) | Yes | For Step 1 (defining criteria for adequate control), the authors mention: “[I]dentifying potential confounders should also rely on content knowledge and theory... Causal diagrams… may be useful in this process, particularly for identifying adjustment variables that are not true confounders and may lead to over- or unnecessary adjustment" and “Causal diagrams also elucidate variables which, if controlled, would theoretically impose (rather than reduce) bias, such as causal intermediates and common effects of the exposure and the outcome”.  For Step 2 (documenting confounder control): “If a study reports multiple adjusted estimates, each controlling for a different set of variables, the abstractors should select the model that best controls for confounding based on the consensus-based criteria from Step 1. For each study, the abstractors document the definition of each adjustment variable, the method for measurement, and how control was achieved. Abstractors should note if studies adjusted for variables not identified as confounders in Step 1, especially if their control could have led to over-adjustment bias.”  For Step 3, the authors mention potential sensitivity analyses excluding studies that controlled for non-confounders. |  | Yes | Proposes a 3-step approach for systematic reviews focused on defining and summarizing confounding control in observational studies and incorporating this assessment into meta-analyses.  “Step 1: define criteria for adequate control of confounding.  Step 2: assess control across component studies using a confounder matrix.  Step 3: incorporate assessment of confounding control into quantitative synthesis for the review.” | Focused on observational studies of aetiology. Focused specifically on confounding - intended to be used alongside other guidance and tools for systematic reviews. |

*Abbreviations.* CASP: Critical Appraisal Skills Programme. COSMOS-E: Conducting Systematic Reviews and Meta-Analyses of Observational Studies of Etiology. CRD: Centre for Reviews and Dissemination. CVD: cardiovascular disease. EPHPP: Effective Public Health Practice Project. GRADE: Grading of Recommendations, Assessment, Development, and Evaluations. JBI: Joanna Briggs Institute. MECIR: Methodological Expectations of Cochrane Intervention Reviews. MMAT: Mixed Methods Appraisal Tool. NICE: National Institute for Health and Care Excellence. NHLBI: National Heart Lung and Blood Institute. NRSI: non-randomised studies of interventions. PRISMA: Preferred Reporting Items for Systematic Reviews and Meta-Analyses. PRISMA-IPD: Preferred Reporting Items for Systematic Reviews and Meta-Analyses of Individual Participant Data. PRISMA-P: Preferred Reporting Items for Systematic Review and Meta-Analysis Protocols. QUIPS: Quality in Prognosis Studies Tool. RAMESES: Realist And MEta-narrative Evidence Syntheses: Evolving Standards. ROB: risk of bias. RoBANS: Risk of Bias Assessment Tool for Non-randomized Studies. ROBINS-I: a tool for assessing Risk of Bias in Non-Randomised Studies of Interventions. ROBINS-E: Risk Of Bias In Nonrandomized Studies - of Exposure tool. RCTs: randomised controlled trials. SES: socioeconomic status. SIGN: Scottish Intercollegiate Guidelines Network. SWIM: Synthesis Without Meta-Analysis.

## References

1. Centre for Reviews and Dissemination (CRD). Systematic Reviews: CRD’s guidance for undertaking reviews in health care 2009. <https://www.york.ac.uk/media/crd/Systematic_Reviews.pdf>. (23 May 2023, date last accessed)

2. Higgins J, Thomas J, Chandler J, et al. *Cochrane Handbook for Systematic Reviews of Interventions version 6.3 (updated February 2022)*. 2022. <https://training.cochrane.org/handbook>. (23 May 2023, date last accessed)

3. Sterne JA, Hernán MA, Reeves BC, et al. ROBINS-I: a tool for assessing risk of bias in non-randomised studies of interventions. *BMJ* 2016; **355**: i4919.

4. Dekkers OM, Vandenbroucke JP, Cevallos M, Renehan AG, Altman DG, Egger M. COSMOS-E: Guidance on conducting systematic reviews and meta-analyses of observational studies of etiology. *PLoS medicine* 2019; **16**: e1002742.

5. Aromataris E, Munn Z. *JBI Manual for Evidence Synthesis*. 2020. <https://synthesismanual.jbi.global>. (23 May 2023, date last accessed)

6. Higgins J, Lasserson T, Chandler J, et al. *Methodological Expectations of Cochrane Intervention Reviews (MECIR) (updated February 2022)*. 2022. <https://community.cochrane.org/mecir-manual>. (23 May 2023, date last accessed)

7. Page MJ, McKenzie JE, Bossuyt PM, et al. The PRISMA 2020 statement: an updated guideline for reporting systematic reviews. *BMJ* 2021; **372**: n71.

8. Page MJ, Moher D, Bossuyt PM, et al. PRISMA 2020 explanation and elaboration: updated guidance and exemplars for reporting systematic reviews. *BMJ* 2021; **372**: n160.

9. Stewart LA, Clarke M, Rovers M, et al. Preferred Reporting Items for a Systematic Review and Meta-analysis of Individual Participant Data: The PRISMA-IPD Statement. *Jama* 2015; **313**: 1657-65.

10. Moher D, Shamseer L, Clarke M, et al. Preferred reporting items for systematic review and meta-analysis protocols (PRISMA-P) 2015 statement. *Syst Rev* 2015; **4**: 1.

11. Shamseer L, Moher D, Clarke M, et al. Preferred reporting items for systematic review and meta-analysis protocols (PRISMA-P) 2015: elaboration and explanation. *BMJ* 2015; **349**: g7647.

12. Wong G, Greenhalgh T, Westhorp G, Buckingham J, Pawson R. RAMESES publication standards: realist syntheses. *BMC Med* 2013; **11**: 21.

13. Campbell M, McKenzie JE, Sowden A, et al. Synthesis without meta-analysis (SWiM) in systematic reviews: reporting guideline. *BMJ* 2020; **368**: l6890.

14. Critical Appraisal Skills Programme (CASP). *CASP Cohort Study and Case Control Study Checklists*. 2018. <https://casp-uk.net/casp-tools-checklists/>. (23 May 2023, date last accessed)

15. Effective Public Health Practice Project (EHPP). *Effective Public Health Practice Project Quality Assessment Tool for Quantitative Studies*. 2010. <https://merst.healthsci.mcmaster.ca/ephpp/>. (23 May 2023, date last accessed)

16. Armijo-Olivo S, Stiles CR, Hagen NA, Biondo PD, Cummings GG. Assessment of study quality for systematic reviews: a comparison of the Cochrane Collaboration Risk of Bias Tool and the Effective Public Health Practice Project Quality Assessment Tool: methodological research. *J Eval Clin Pract* 2012; **18**: 12-8.

17. Thomas BH, Ciliska D, Dobbins M, Micucci S. A process for systematically reviewing the literature: providing the research evidence for public health nursing interventions. *Worldviews Evid Based Nurs* 2004; **1**: 176-84.

18. Hong QN, Pluye P, Fàbregues S, et al. Improving the content validity of the mixed methods appraisal tool: a modified e-Delphi study. *J Clin Epidemiol* 2019; **111**: 49-59.e1.

19. National Institutes of Health National Heart LaBI. *Quality Assessment Tool for Observational Cohort and Cross-Sectional Studies and Quality Assessment of Case Control Studies*. 2013. <https://www.nhlbi.nih.gov/health-topics/study-quality-assessment-tools>. (23 May 2023, date last accessed)

20. National Institute for Health and Care Excellence (NICE). *Methods for the development of NICE public health guidance (third edition)*. 2012. <https://www.nice.org.uk/process/pmg4/chapter/appendix-g-quality-appraisal-checklist-quantitative-studies-reporting-correlations-and>. (23 May 2023, date last accessed)

21. Hayden JA, Windt DAvd, Cartwright JL, Cote P, Bombardier C. Assessing Bias in Studies of Prognostic Factors. *Ann Int Med* 2013; **158**: 280-6.

22. Kim SY, Park JE, Lee YJ, et al. Testing a tool for assessing the risk of bias for nonrandomized studies showed moderate reliability and promising validity. *J Clin Epidemiol* 2013; **66**: 408-14.

23. ROBINS-E Development Group. *Risk Of Bias In Nonrandomized Studies - of Exposure (ROBINS-E). Launch version, 1 June 2022*. 2022. <https://www.riskofbias.info/welcome/robins-e-tool>. (23 May 2023, date last accessed)

24. Scottish Intercollegiate Guidelines Network (SIGN). *Methodology Checklist 3: Cohort Studies (Version 3). Methodology Checklist 4: Case-Control Studies (Version 2)*. 2012. <https://www.sign.ac.uk/what-we-do/methodology/checklists/>. (23 May 2023, date last accessed)

25. Petersen JM, Barrett M, Ahrens KA, et al. The confounder matrix: A tool to assess confounding bias in systematic reviews of observational studies of etiology. *Res Synth Methods* 2022; **13**: 242-54.
